# Biodiesel production from alternative raw materials using a heterogeneous low ordered biosilicified enzyme as biocatalyst

**DOI:** 10.1186/s13068-021-01917-x

**Published:** 2021-03-15

**Authors:** Gabriel Orlando Ferrero, Edgar Maximiliano Sánchez Faba, Griselda Alejandra Eimer

**Affiliations:** CITeQ-UTN-CONICET, Facultad Regional Córdoba, Universidad Tecnológica Nacional, Maestro López Esq. Cruz Roja, Ciudad Universitaria, 5016, Córdoba, Argentina

**Keywords:** Enzymatic biosilicification, Second-generation biodiesel, Alternative oils, Mesoporous material, *Pseudomonas fluorescens* lipase

## Abstract

**Background:**

Cumulative reported evidence has indicated that renewable feedstocks are a promising alternative source to fossil platforms for the production of fuels and chemicals. In that regard, the development of new, highly active, selective, and easy to recover and reuse catalysts for biomass conversions is urgently needed. The combination of enzymatic and inorganic heterogeneous catalysis generates an unprecedented platform that combines the advantages of both, the catalytic efficiency and selectivity of enzymes with the ordered structure, high porosity, mechanical, thermal and chemical resistance of mesoporous materials to obtain enzymatic heterogeneous catalysts. Enzymatic mineralization with an organic silicon precursor (biosilicification) is a promising and emerging approach for the generation of solid hybrid biocatalysts with exceptional stability under severe use conditions. Herein, we assessed the putative advantages of the biosilicification technology for developing an improved efficient and stable biocatalyst for sustainable biofuel production.

**Results:**

A series of solid enzymatic catalysts denominated LOBE (low ordered biosilicified enzyme) were synthesized from *Pseudomonas fluorescens* lipase and tetraethyl orthosilicate. The microscopic structure and physicochemical properties characterization revealed that the enzyme formed aggregates that were contained in the heart of silicon-covered micelles, providing active sites with the ability to process different raw materials (commercial sunflower and soybean oils, *Jatropha excisa* oil, waste frying oil, acid oil from soybean soapstock, and pork fat) to produce first- and second-generation biodiesel. Ester content ranged from 81 to 93% wt depending on the raw material used for biodiesel synthesis.

**Conclusions:**

A heterogeneous enzymatic biocatalyst, LOBE4, for efficient biodiesel production was successfully developed in a single-step synthesis reaction using biosilicification technology. LOBE4 showed to be highly efficient in converting refined, non-edible and residual oils (with high water and free fatty acid contents) and ethanol into biodiesel. Thus, LOBE4 emerges as a promising tool to produce second-generation biofuels, with significant implications for establishing a circular economy and reducing the carbon footprint.

## Background

Processing biomass to produce fuels and chemicals is often impractical due to the high costs of materials, the number of purification steps required, low selectivity, and other current technological limitations. Biodiesel derived from vegetable oils or animal fats is a renewable substitute for diesel fuel. Current research has focused on new biodiesel production and purification technologies that aim to reach a carbon-neutral footprint as well as to use cheap, renewable and abundant raw materials not used for food production [1]. In this context, residual (waste frying oils), non-edible (*Jatropha* oils), and other vegetable oils (acid oil from soybean soapstock), which have no value as food, arise as alternative feedstock since their energy content can be used as raw material for second-generation fuel production [2–4]. In fact, these oils could be used leaving aside the controversy about oils for food and fuels and promoting a circular economy. Thus, it is necessary to consider novel synthesis routes or process technologies for the valorization of oils and promoting their efficient use, reducing the processing time, and employing the principles of green chemistry and sustainable development [5, 6]. Over the past decades, enzymes were established as a new class of catalysts in the field of modern synthetic chemistry [7–9]. They have been continuously gaining importance due to the discovery of new applications and the development of much more efficient production systems. Consequently, the application of enzymes as biocatalysts in industrial processes has emerged as an attractive strategy to avoid cumbersome design and synthesis of artificial catalysts [7–12]. Several reasons underlie the rapid evolution of these biological catalysts, including their high catalytic efficiency and selectivity (stereo-, regio-, and chemo-selectivity). Moreover, the enzymatic reaction avoids the use of protecting groups and minimizes side reactions in mild operating conditions, thus reducing environmental constraints. However, such practical applications are hindered by the fragile nature of enzymes, such as low thermal stability (reduced half-live at temperatures over 70 °C [13]), narrow optimum pH for activity (pepsin: 2, acid phosphatase: 5.7, and alkaline phosphatase: 10.5 [14]), low tolerance to most organic solvents (chymotrypsin activity decreases at the threshold concentrations of 23% and 33% v/v of 1-propanol and 2-propanol, respectively [15]; polyphenol oxidase and trypsin activities are reduced when the solution has a concentration of 50% v/v of tetrahydrofuran, dioxane, acetone, or acetonitrile [16]), and salt concentration in the medium (increased ionic strength decreases the *S. solfataricus* carboxypeptidase activity [17]). To overcome these limitations in the use of enzymes in biocatalytic systems, immobilization of proteins on mesoporous silica supports has been reported as a promising strategy [18–21]. Mesoporous silica materials have unique advantages including an ordered structure with high porosity and specific surface, which offer convenient mechanical, thermal and chemical resistance. The pore system in the order of 2 nm to 5 µm allows molecular discrimination according to size, and the diffusion of substrates and products. Moreover, pore size can be adjusted to match that of a given enzyme; likewise, pore shape can be ordered differently depending on the synthesis conditions, such as hexagonal or cubic [22, 23], allowing enzyme loading and also providing a protective environment where the enzymes can often tolerate extreme pH, high temperatures and salt concentrations [24–26]. The surface of mesoporous solids can be modified to increase their hydrophobic or hydrophilic behavior, or to improve their activity by incorporating different metals as catalytically active species [23, 27, 28]. Such features enhance enzyme stability and function (with a synergistic effect between the enzyme and the support), improving the reaction yields. On the other hand, they can simplify the processes of biocatalyst recovery and reuse, and purification of the products [25, 29, 30].

Co-precipitation is one of the commonly used methods to immobilize enzymes, in which proteins are immobilized on a support during its synthesis: a silicon organic precursor (tetraethyl orthosilicate, TEOS) is added to a mixture of dodecylamine (surfactant), acetonitrile (organic solvent) and a buffer solution [31]. This process of enzymatic mineralization is denominated biosilicification and provides an exceptional biocatalyst stability under drastic conditions [31–33]. According to Cebrián-García et al., the conversion of valeric acid and ethanol to ethyl valerate is maintained over 93% efficiency in the presence of biosilicified *Candida antarctica* lipase B (Cal-B) [34]. Besides, Itabaiana et al. described that the absence of organic solvents or low enzyme concentration in the buffer during the biocatalyst synthesis produces materials without microporous structure and with low activity [33]. They employed the biosilicified Cal-B enzyme to esterify fatty acids under continuous flow conditions. The encapsulated enzyme showed improved activity when compared with the free enzyme, as well as with the commercial Novozyme strain. Moreover, Pistone et al. reported that they succeeded in immobilizing different laccases by silica mineralization in one-pot process, modifying the immobilization conditions (pH, organic solvent, enzyme content, etc.) [32]. Although the oxidation rates of the biocatalysts were lower than those of the free enzymes, the biosilicified enzymes showed to be highly stable and reusable [32].

In the present work, we applied the advantages of the biosilicification technology for the design of an efficient and stable biocatalyst for sustainable biodiesel production. A low ordered biosilicified enzyme (LOBE) was successfully synthesized in only one-step reaction using a lipase enzyme from *Pseudomonas fluorescens* and TEOS as silicon source. In that way, a biocatalyst with mesoporous structure and the ability to produce biodiesel from different oils and commercial ethanol as raw materials was obtained. The synthesis conditions and the characterization of the biocatalyst, together with the enzymatic performance, are described in detail.

## Results and discussion

### Synthesis and evaluation of biosilicified* Pseudomonas fluorescens* lipase

Based on previous reports [31–33, 35], biosilicified *Pseudomonas fluorescens* lipases were synthesized with different theoretical amounts of enzyme (0, 1, 5, 10, 50, 100 and 150 mg) and the activity of the obtained biocatalysts to produce biodiesel was evaluated.

Initial essays were carried out employing the encapsulated lipases as biocatalysts, and sunflower oil and food-, pharmaceutical-, and cosmetic-grade ethanol (96 v/v% and available in most local grocery stores) as raw materials. The latter was chosen for three reasons: (1) the enzyme needs the water to be active (4% v/v H_2_O is adequate; the water acts as a lubricant allowing the enzyme active site to open and interact with the substrates) [22, 25, 36]; (2) it is produced from renewable sources, and (3) ethanol has lower flammability and toxicity than methanol.

**Fig. 1:**
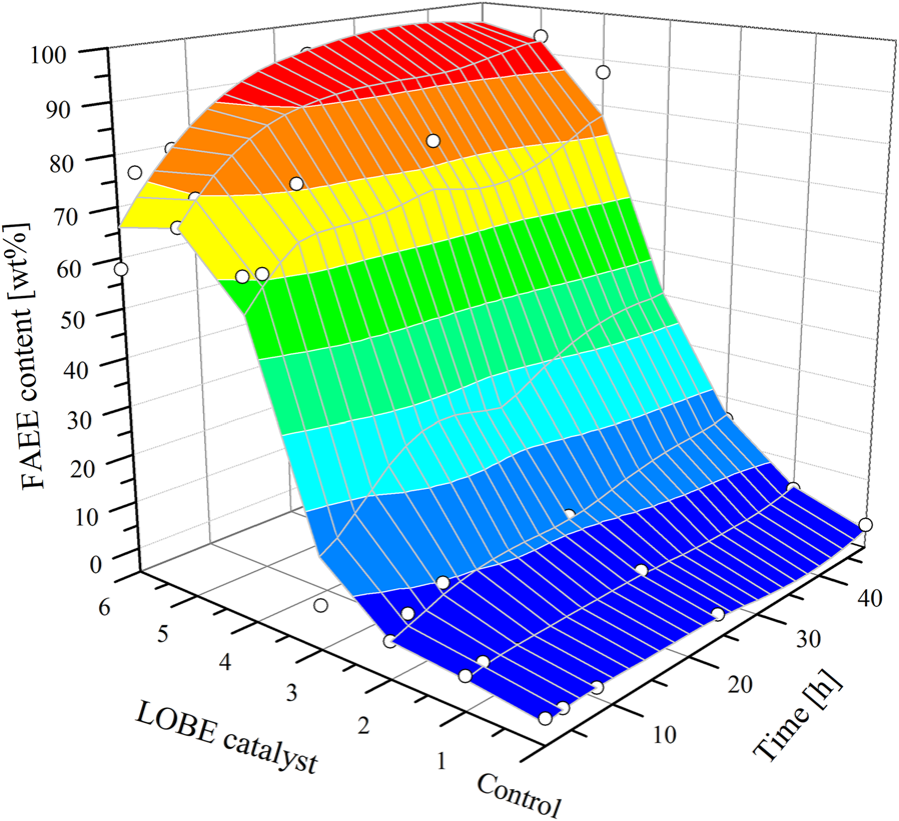
FAEE content versus enzyme loading (in all the LOBE catalysts) and reaction time

Transesterification activity was detected for all biocatalysts analyzed (Fig. 1). Moreover, the activity of each biocatalyst increased together with the amount of immobilized enzyme. However, only protein loadings higher than 67 mg_protein_/g_support_ produced acceptable fatty acid ethyl esters (FAEE) contents (Table 1). Moreover, the immobilization efficiency of lipases achieved during the biosilicification process was higher than 90% with respect to the theoretical content. On the other hand, biodiesel production was measured under initial rate conditions (2 h in our system and the enzymatic reaction showing linear behavior) in order to compare the different FAEE contents obtained. As can be observed, with the sole exception of LOBE6, the FAEE content showed to be proportional to the lipase content (Table 1).

**Table 1:** Protein loading, FAEE content and specific activity of the synthesized biocatalysts

Biocatalyst	Theoretical protein amount (mg)	Protein loading (mg_protein_/g_support_)	Immobilization efficiency (%)	FAEE content (wt%)	Specific activity (U/mg_lipase_)
Free lipase	50	–	–	30.17	5.03
LOBE1	1	1.17	98.60	0.45	25.59
LOBE2	5	6.04	99.70	2.43	26.97
LOBE3	10	11.80	99.82	3.87	22.12
LOBE4	50	66.85	96.88	62.39	66.37
LOBE5	100	120.66	97.66	68.61	42.48
LOBE6	150	141.72	96.03	57.82	31.05

Based on FAEE content results, LOBE5 appeared to have the best biocatalytic activity. However, when the specific activity was determined, LOBE4 showed to have the best performance: the specific activity was 57% higher than that of LOBE5. Therefore, LOBE4 was chosen to proceed with its physicochemical characterization.

It should be highlighted that when the free lipase was used, significantly fewer contents of FAEE were produced (Table 1). That was probably since free enzyme forms aggregates in the organic reaction mixture, which reduces the number of exposed active catalytic sites, and therefore, making it less efficient [25].

As control, when material synthesis in the absence of enzyme was evaluated, no FAEE production was detected, indicating that the lipase is responsible for the transesterification activity.

### Physicochemical characterization of the LOBE4 biocatalyst

**Fig. 2:**
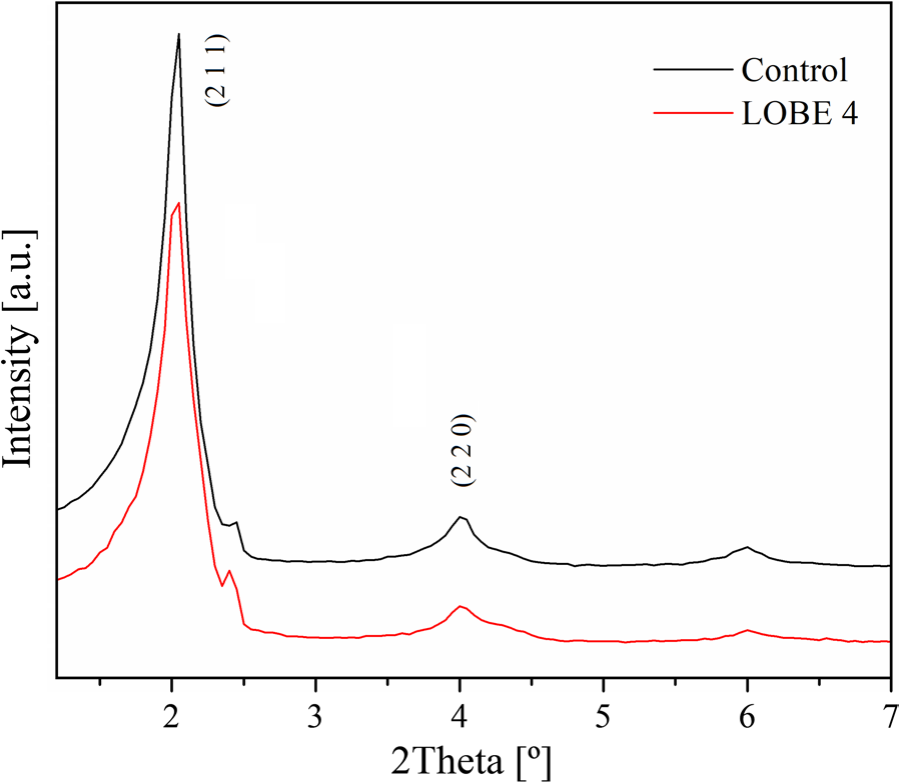
X-ray diffractions patterns of control and LOBE4

**Scheme 1:**
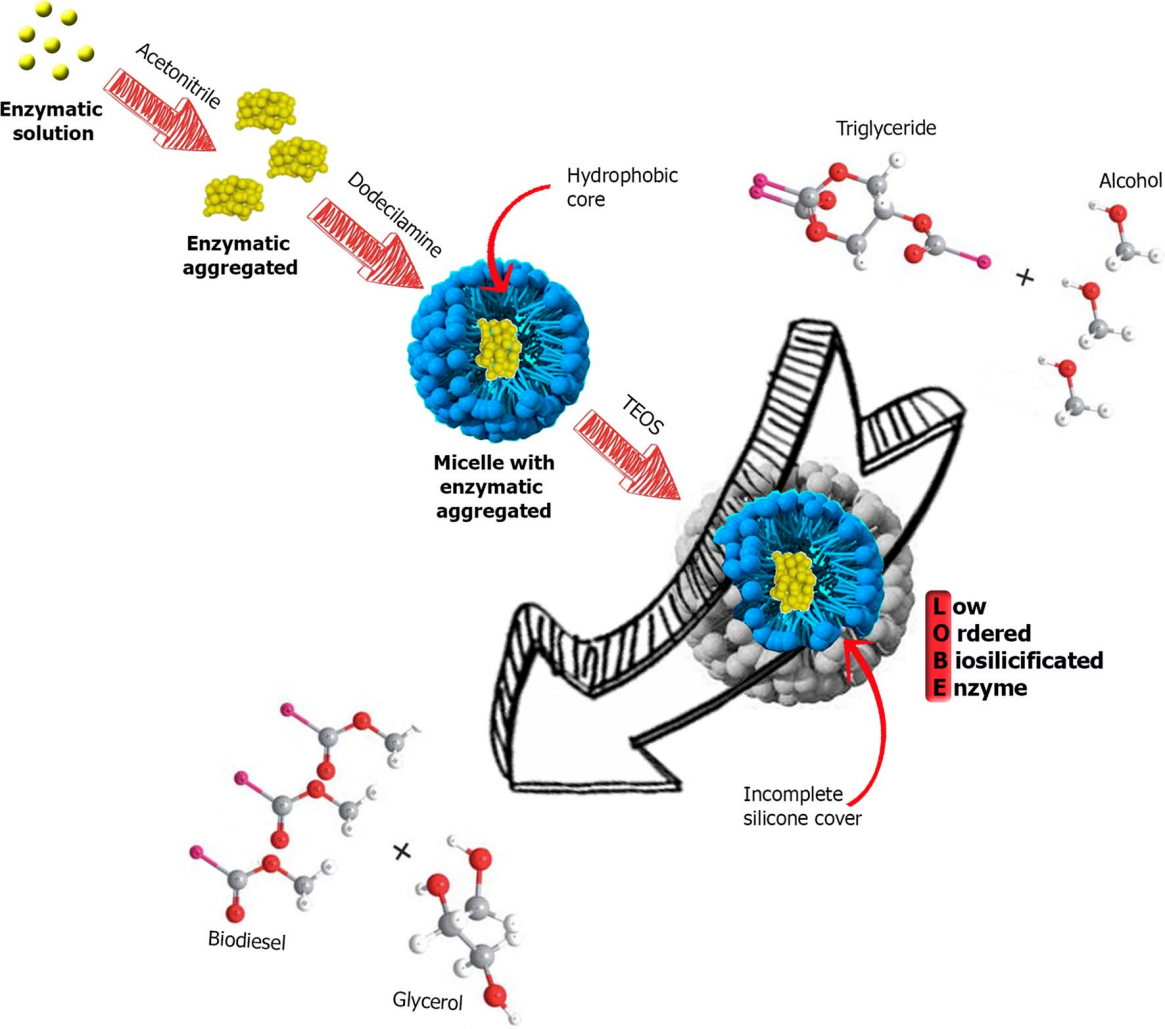
Proposed LOBE synthesis mechanism

The structural and textural properties of the biosilicified enzymes were studied by X-ray diffraction (XRD), transmission electron microscopy (TEM), N_2_ adsorption/desorption to obtain the specific areas by the BET method and Fourier transform infrared spectroscopy. The typical ordering of mesoporous silicates was detected when the control and LOBE4 were analyzed by small angle XRD. As shown in Fig. 2, experimental spectra exhibit two maxima peaks which can be assigned to the typical (2 1 1) and (2 2 0) diffraction planes of a three-way pore structure such as that of Mobil Composition of Matter No. 48 (MCM-48) siliceous solid. In addition, the ratio value d_220_/d_211_ was approximately 0.87, which is also in accordance with the cubic structure of MCM-48 and indicates a structural organization in the architecture of biosilicified enzymes [37, 38]. *n*-Dodecylamine (DDA) is a molecule with a polar head and a hydrophobic chain, which can form micelles and act as a surfactant. In solution, DDA gives rise to a liquid crystal micellar phase that can be employed as structure directing agent to form mesoporous structures (such as MCM-48) when TEOS is mineralized [35, 37, 39, 40]. During this process, the enzyme can insert itself into the micelles without interfering with the formation of the cubic phase (Scheme 1).

**Fig. 3:**
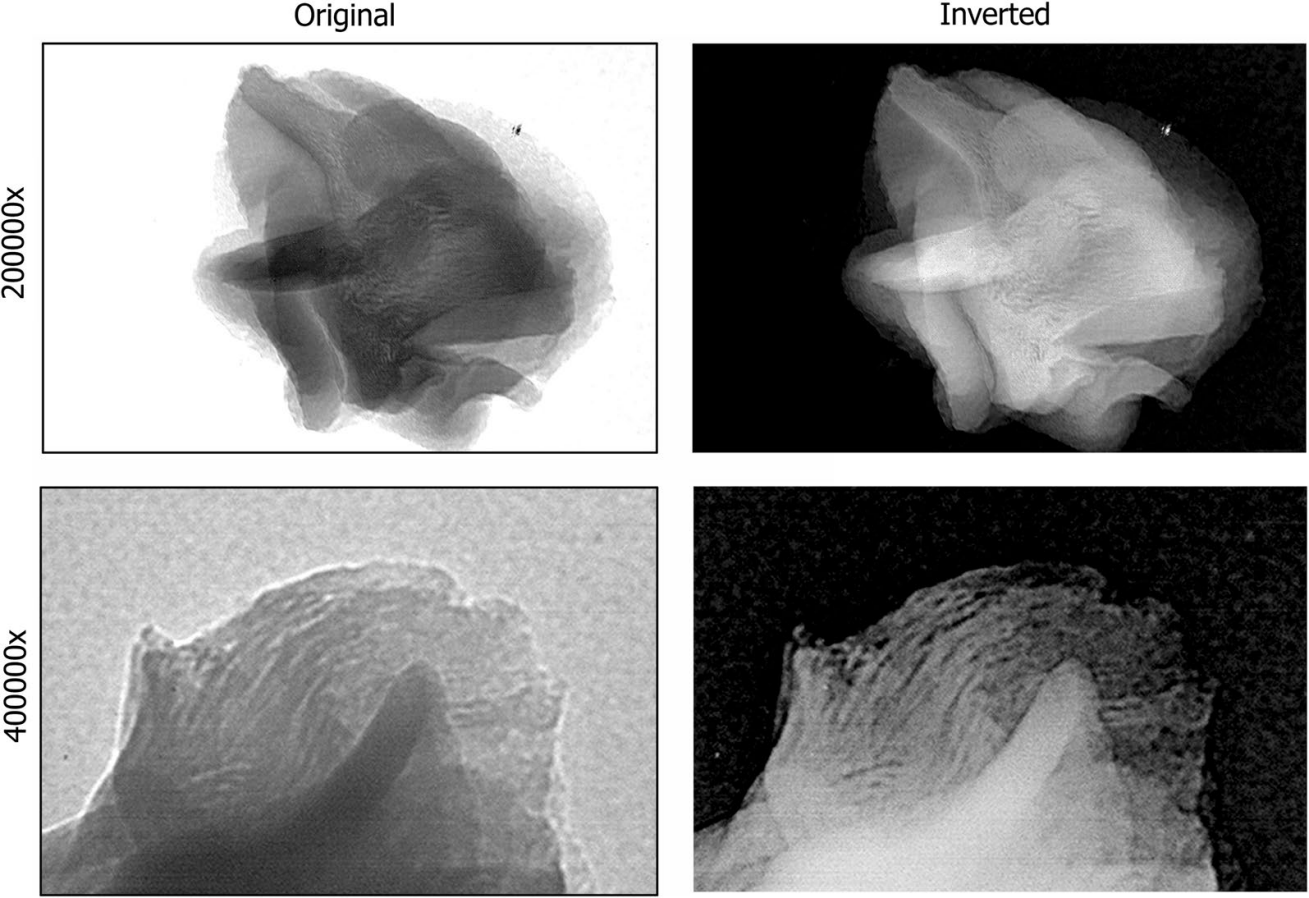
TEM images of LOBE4 biocatalyst structure

As can be observed in Fig. 3, TEM analysis revealed that LOBE 4 has a nanotubular structure like a nanofiber network with channels. Similar results were previously reported by Garcia et al., indicating that the siliceous mineralization has been carried out over the enzyme (to facilitate the observation of nanofiber network architecture, LOBE4 images are showed in original and inverted colors) [31]. Noteworthy, and as shown in Fig. 1, the biosilicification process does not affect the activity of the enzymes or the flow of substrates and products.

**Fig. 4:**
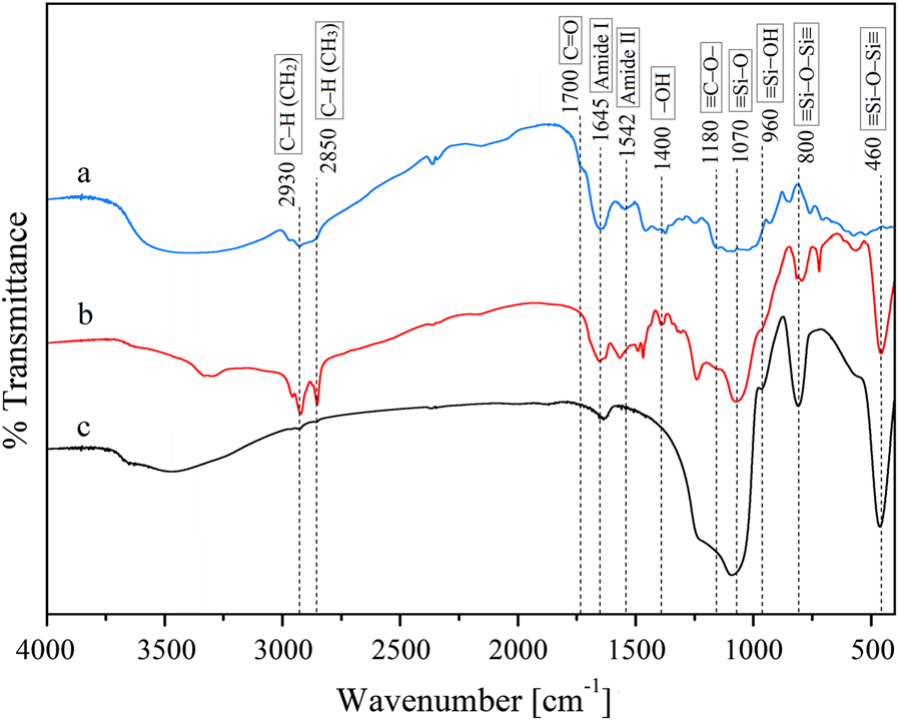
FT-IR analysis of biocatalysts: **a** free *Pseudomonas fluorescens* lipase, **b** LOBE4 and **c** calcined LOBE4

Infrared spectroscopy is a widely used technique to study the substructure of peptides and proteins and it can be used to monitor the presence of the proteins on the supports [41, 42]. Figure 4a shows the different functional groups characteristic of the free *Pseudomonas fluorescens* lipase evidenced by FT-IR. The stretching C–H vibrations of –CH_2_ and –CH_3_ groups, the stretching vibrations of carbonyl groups, and stretching vibration of ≡C–O– groups were detected. The signal of the –OH deformation vibrations appears at 1400 cm^−1^ [43]. Finally, the signals for most characteristic functional groups of the pure enzymes, amide I and amide II, were also observed (Fig. 4a) [44, 45]. The decrease of the intensities and wavenumbers of amide bands in LOBE4 FT-IR spectrum indicates that the immobilization of the lipase inside the silica matrix was successful (Fig. 4b) [46]. Furthermore, the presence of the amide I and amide II bands evidences that the secondary structure and bioactivity of the enzyme are conserved when the protein is incorporated into the nanostructure (Fig. 1) [47]. The presence of silicon could be corroborated by the ≡Si–O bond vibration band [48–50]. Bands between 3500 and 3300 cm^−1^ correspond to primary amine (–N–H bond) of DDA (Fig. 4b). Additionally, the bands assigned to C–H stretching of the saturated –CH_2_ and –CH_3_ groups increase their intensity in comparison to the free enzyme, due to the surfactant presence in the biocatalyst (Fig. 4b). These bands disappeared when the biocatalyst was calcined at 500 °C for 8 h, indicating that the organic material was removed (Fig. 4c).

The FT-IR spectrum of the calcined biocatalyst reveals only the characteristic bands of the siliceous matrix such as the ≡Si–O bond vibration, the ≡Si–OH bending, and ≡Si–O–Si≡ symmetric stretching and bending (Fig. 4c) [48–50, 51].

Since the FT-IR spectrum of LOBE4 showed the presence of typical functional groups of the enzyme and the siliceous material, it confirmed the effective immobilization of lipase through biosilicification. Then, control material and LOBE4 (calcined and non-calcined) specific surface was determined (Table 2).

**Table 2:** Specific areas of control and LOBE4 before and after calcination.

Sample	Specific areas (m^2^/g ^(C)^)	Specific areas (m^2^/g^(NC)^)
Control	221.01	4.97
LOBE4	289.54	6.12

C: calcined; NC: non-calcined

**Fig. 5:**
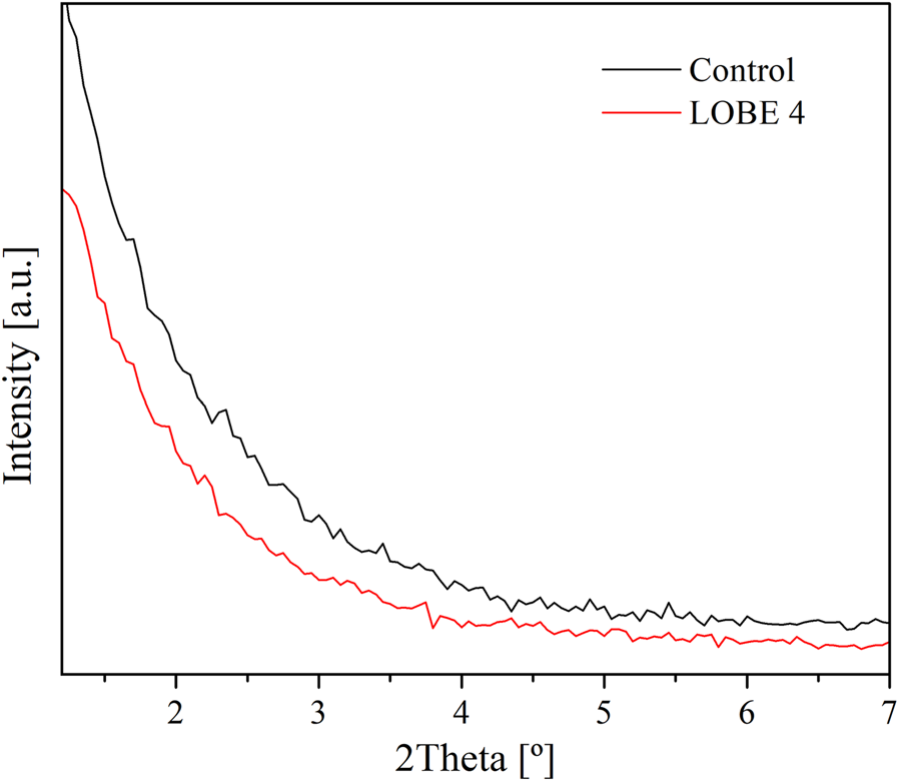
X-ray diffractions of calcined control and LOBE 4

After calcination at 500 °C, the specific area was expected to increase due to the elimination of the organic phase (surfactant and lipase). Nevertheless, the XRD pattern of the calcined sample did not show the permanence of an ordered structure (Fig. 5), indicating that the architecture of the siliceous network generated by the biosilicification process is unstable at high temperatures.

These results indicate the formation of an ordered but incomplete siliceous–organic hybrid structure, further suggesting that silicification fails to cover the entire organic structure. Consequently, by removing the organic template, the nanostructure collapses. Nonetheless, the incomplete mineralization gives the biocatalyst a certain flexibility that allows the diffusion of substrates and products to the active sites of the enzymes. Due to these characteristics, these materials were denominated as low ordered biosilicified enzyme (LOBE) (Scheme 1).

### Assessment of LOBE4 activity with alternative raw materials

**Fig. 6:**
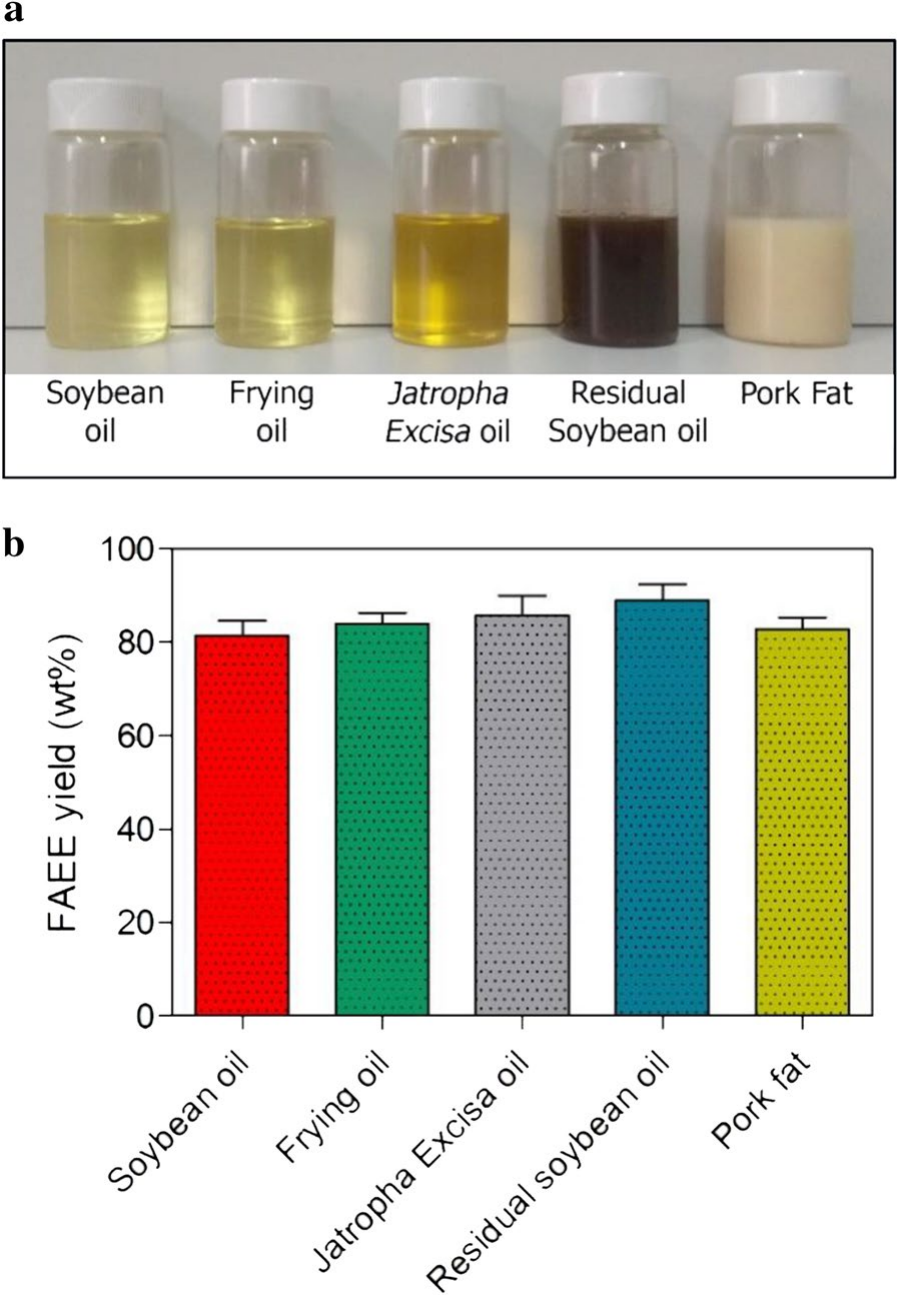
Performance of LOBE4 biocatalyst. **a** Used feedstocks. **b** Obtained FAEEs contents from the different raw materials used. Reaction conditions: ethanol/oil molar ratio = 4/1; 125 mg of LOBE4/g of oil, 37 °C, reaction time = 24 h, constant shaking (80 oscillations/min)

To analyze the versatility of LOBE4, its capacity to catalyze the production of biodiesel was tested with the following feedstocks: soybean oil, waste frying oil, *Jatropha excisa* oil, acid oil from soapstock and pork fat (Fig. 6a). The selection of these raw materials was based on the reasons detailed below. In 2018, Argentina was the world leading exporter of soybean oil and the world third largest exporter of sunflower oil [52]. Besides, the use of soybean oil as raw material for the production of biodiesel does not produce a detrimental effect on the local population food availability since sunflower oil is mostly consumed in Argentina. Furthermore, biocatalytical conversion of soybean oil into fuels and chemicals of commercial interest would foster local industrialization and employment generation [53]. In addition, the process of soybean oil purification generates an acid oil side-product containing a large amount of free fatty acids (50-80wt% of FFA approximately) and, to a lesser extent, a mixture of phospholipids, tocopherols, sterols, degraded and oxidized residues, pigments, salts, color bodies, triglycerides, diglycerides and monoglycerides [54, 55]. Converting this acid oil into biodiesel represents an attractive added value strategy for a more efficient use of agricultural production and recycling.

Moreover, waste frying oils are a domestic and gastronomic industry waste with high energy content. These waste oils are available in large quantities at a minimal cost and are often discarded in drains, causing obstructions in the sewer system and polluting water resources. Recycling them for the production of biodiesel could be a greener alternative to substantially reduce the price of biofuel [56–58].

In addition, the use of non-edible oilseeds for the production of biofuel is also an attractive alternative as a raw material in place of oils intended for food. *Jatropha excisa* is an endemic and non-conventional oilseed species from the semiarid and arid northwest region of Argentina with an average oil concentration of 34 wt%. Although this oil is presumably toxic, native people have used it for centuries in traditional medicine as purgative and emetic [59]. It does not represent a competition for agricultural food crops and diversifies farmland, emerging as an alternative feedstock for biofuel production with high economic potential [60, 61].

Animal fats are other alternative raw material source for biodiesel production since their cost is considerably lower than that of vegetable oils. These feedstocks are currently added to pet food and animal feed or used for industrial soap production. Nevertheless, many researches have shown that these raw materials can effectively be transformed into a high-quality biodiesel that meets the ASTM specifications [63, 64].

Excluding soybean oil, the remaining raw materials detailed represent interesting sources for the production of second-generation biofuels. Nevertheless, their high content of free fatty acids and water does not allow their direct use in the conventional homogenous process of biodiesel production (Table 3). In turn, free fatty acids must be esterified with sulfuric acid and methanol (homogeneous acid process); then, the acid catalyst in solution must be neutralized, and the product should be washed and dried. Only after this pretreatment, the resulting raw material (a mixture of fatty acids esters and triglycerides) can be employed in the transesterification reaction with a homogeneous basic catalyst. Finally, neutralization, washing and drying steps must be performed again to obtain the product that will be used as fuel [65, 66]. All these steps substantially increase the cost of the process.

**Table 3:** Characterization of raw materials

Feedstock	Density (g/cm^3^)	Kinematic viscosity (mm^2^/s) ^a^	Acid value (mg_KOH_/g_oil_)	FFA content (wt%) ^b^	Water content (ppm)	Triglyceride content (wt%)
Soybean oil	0.93	18.38	0.13	0.07	626	97.70
Waste Frying oil	0.94	20.48	0.21	0.11	671	95.73
*Jatropha excisa* oil	0.92	15.75	1.55	0.78	980	94.60
Acid oil from soybean soapstock	0.96	10.94	153.72	76.91	5221	9.18
Pork Fat	0.90	21.85	0.67	0.33	590	97.64

^a^ At 60 °C (reaction temperature)

^b^ Calculated from the acid value (EN 14104: 2003) and expressed as oleic acid [62]

As shown in Fig. 6b, the five oily raw materials analyzed were successfully converted into biodiesel in the presence of ethanol and LOBE4, which indicates that the biocatalyst was able to mediate the transesterification of acylglycerols and the esterification of the FFA without any previous treatment. Therefore, when the reaction is complete, purification steps are reduced to just separating the biocatalyst from the reaction mixture by simple filtration and removing excess ethanol (which can be also recovered and reused in the next reaction). Then, the biocatalyst can be reactivated by washing with acetone (to eliminate the organic residues) and dried at room temperature to be reused in the next cycle. Overall, the sustainability of this process lies in the use of alternative, renewable, and low-cost raw materials to produce second-generation biofuels that reduce the emission of carbon dioxide.

## Conclusions

In this work, a heterogeneous enzymatic biocatalyst was developed in a single-step synthesis reaction according to the following proposed mechanism: first, enzymes form aggregates when mixed with the organic solvent (acetonitrile), which would leave their hydrophobic groups exposed to the solvent. By adding the surfactant, these enzymatic aggregates could be inserted into the DDA micelles while maintaining their agglomerated structure due to the hydrophobic core of the micelles. In other words, enzymes remain grouped without the need to perform a non-specific crosslinking to keep them together, which would affect their activity. Then, when this molecular macrostructure is covered with an incomplete silicon network, a solid hybrid (enzyme–inorganic) catalyst is obtained. The concentration of active sites of the aggregated enzymes and the flexibility of the incomplete silicon network allows the diffusion of substrates and products through the *low ordered biosilicified enzyme* (LOBE). This hybrid structure can catalyze the production of first- and second-generation biodiesel from a variety of oils (edible, non-edible, residual oils) and animal fats.

## Methods

### Materials

*Pseudomonas fluorescens* lipase (PFL, ≥ 20.000 IU/g at 55 °C, pH 8.0) was purchased from Sigma-Aldrich Co. (St. Louis, USA). Sunflower oil (Cocinero, Molinos Río de la Plata S.A., Argentina), soybean oil (Sojola, Aceitera General Deheza S.A., Argentina), pork fat (Paladini S.A., Argentina) and ethanol 96% v/v (Porta Hnos. S.A., Argentina), were purchased at local grocery stores. Waste frying oil was obtained from different domestic sources and filtered before being used. Acid oil from soapstock (soybean) was generously gifted by a local company (Louis Dreyfus Company, Argentina). *Jatropha excisa* oil was kindly donated by Dr. Fracchia (CRILAR-CONICET, La Rioja, Argentina). Other used reagents were: KH_2_PO_4_, K_2_HPO_4_ and KOH (Anedra), n-dodecylamine (Sigma-Aldrich, USA), n-hexane (analytical grade, Merck), isopropyl alcohol (Fluka), acetonitrile (analytical grade, Merck), tetraethyl orthosilicate (Aldrich) and milliQ water. Syringe filters (polypropylene, 25 mm diameter and 0.2 micron pore size) were supplied by VWR (USA).

### Acid value determination

Feedstocks acid values were determined by volumetric titration according to the standard EN ISO 14104 (2003). The required oil mass was mixed with 2-propanol in a conical flask (0.25 g_sample_/mL_solvent_), and titrated using a KOH 0.1M aqueous solution. Phenolphthalein was used as the final point indicator. Results are expressed in mg_KOH_/g_sample_.

### Pseudomonas fluorescens lipase biosilicification

Different lipase amounts (0.00–150.00 mg) were dissolved with 6.36 mL of 50 mM phosphate buffer (pH = 7.5). Then, 6.36 mL of acetonitrile and 0.625 mL of *n*-dodecylamine were added to the solution while stirring. Finally, 1 mL of tetraethoxysilane was added to the mixture. After a few minutes, a visibly solid precipitate formed, and the suspension was stirred during 3 h at 25 °C. After that, the obtained solid was filtered, washed with water and dried at room temperature for 24 h. Protein content quantification was performed in the liquid supernatant (“supernatant protein”) by the Bradford method [67]. The hybrid material obtained from the enzymatic immobilization was named as LOBE X, where X is related with the enzyme content.

The amount of immobilized lipase was reported as “Protein Loading” and was determined according to the following equations:

$$\text{Actual protein }({\text{mg}}_{\text{protein}})=\text{Theoretical protein }\left(\text{mg}\right)-\text{ Supernatant protein }(\text{mg})$$

$$\text{Protein loading }\left(\frac{{\text{mg}}_{\text{protein}}}{{\text{g}}_{\text{support}}}\right)=\frac{\text{Actual protein }\left(\text{mg}\right)}{\text{Support }\left(\text{g}\right)}$$

Immobilization efficiency was calculated as follows:

$$\text{Immobilization efficiency }(\text{\%}) =\frac{\text{Actual protein }\left(\text{mg}\right)}{\text{Theoretical protein mg}) }\times 100.$$

### Support characterization

The X-ray diffraction (XRD) patterns were recorded in air at room temperature on a PANalytical X-Pert Pro X-ray powder diffractometer, with a Bragg–Brentano geometry. A CuKα lamp was used (40 kV, 40 mA) in 2*Ɵ* range between 1.5 and 7°. The interplanar distance (d_100_) was estimated using the position of the first X-ray diffraction peak. BET method was employed to measure the specific surface of the materials. A Micrometrics Pulse ChemiSorb 2700 equipment was used. The samples were previously heated for 1 h at 300 °C under N_2_ flow. FT-IR analyses were performed on a Thermo Scientific Nicolet iS10 spectrometer, with Smart OMNI-Transmission accessory. The measure range was from 400 to 4000 cm^−1^ with a resolution of 4 cm^−1^ and 50 scans. Samples were prepared by the KBr technique.

### Transesterification reaction

Reactions were carried out in glass screw vials placed in an orbital shaker at 80 rpm and 37 °C. The used oil/ethanol molar ratio was 1/4. Reactions started when the biocatalysts were added (125 mg_biocatalyst_/g_oil_). Samples were taken at different times and were analyzed by high-performance liquid chromatography (HPLC) [25]. The “Enzymatic activity” and the “Specific activity” were calculated according to the following equations:

$${\text{Enzymatic activity }(\text{U}) }=\frac{\text{Produced FAEE content }(\text{mg})}{\text{ Time }(\text{min})},$$

$$\text{Specific activity }\left(\frac{\text{U}}{{\text{mg}}_{\text{protein}}}\right)=\frac{\text{U}}{\text{ Actual protein }\left(\text{mg}\right)}.$$

### Chromatographic analysis

Analyses were performed with a Perkin Elmer HPLC with UV–Vis detector of the 200 series equipped with a solvent delivery unit with gradient of elution, a KNAUER Vertex Plus column (250 mm × 4.6 mm, 5 μm) Eurospher II 100–5 C18 P and TotalChrom software. The UV detector wavelength was set at 205 nm, the column temperature was maintained at 30 °C, and the flowrate was 1 mL/min. For chromatographic runs, a stepwise method was used: 100% of methanol in 0 min, 50% of methanol and 50% of 5:4 2-propanol/n-hexane in 10 min, maintained with isocratic elution for 10 min [68]. All reactions were performed at least in duplicate and the results were expressed as mean values, being percentage differences between them and the mean always less than 5%.

## Data Availability

All data generated or analyzed during this study are included in the manuscript.
